# Dosimetric characterization of the M−15 high‐dose‐rate Iridium−192 brachytherapy source using the AAPM and ESTRO formalism

**DOI:** 10.1120/jacmp.v16i3.5270

**Published:** 2015-05-08

**Authors:** Minh‐Tri Ho Thanh, John J. Munro, David C. Medich

**Affiliations:** ^1^ Department of Physics Worcester Polytechnic Institute Worcester MA USA; ^2^ Source Production Equipment Company Inc. St. Rose LA USA

**Keywords:** HDR brachytherapy, Ir‐192, TG‐43U1

## Abstract

The Source Production & Equipment Co. (SPEC) model M−15 is a new Iridium−192 brachytherapy source model intended for use as a temporary high‐dose‐rate (HDR) brachytherapy source for the Nucletron microSelectron Classic afterloading system. The purpose of this study is to characterize this HDR source for clinical application by obtaining a complete set of Monte Carlo calculated dosimetric parameters for the M‐15, as recommended by AAPM and ESTRO, for isotopes with average energies greater than 50 keV. This was accomplished by using the MCNP6 Monte Carlo code to simulate the resulting source dosimetry at various points within a pseudoinfinite water phantom. These dosimetric values next were converted into the AAPM and ESTRO dosimetry parameters and the respective statistical uncertainty in each parameter also calculated and presented. The M−15 source was modeled in an MCNP6 Monte Carlo environment using the physical source specifications provided by the manufacturer. Iridium−192 photons were uniformly generated inside the iridium core of the model M−15 with photon and secondary electron transport replicated using photoatomic cross‐sectional tables supplied with MCNP6. Simulations were performed for both water and air/vacuum computer models with a total of $4\times 10^{9}$ sources photon history for each simulation and the in‐air photon spectrum filtered to remove low‐energy photons below δ=10%keV. Dosimetric data, including $D(r,\theta ),g_{L}(r),F(r,\theta ),\Phi_{\text{an}}(r)$, and ${\bar{\varphi }}_{\text{an}}$, and their statistical uncertainty were calculated from the output of an MCNP model consisting of an M−15 source placed at the center of a spherical water phantom of 100 cm diameter. The air kerma strength in free space, $S_{K}$, and dose rate constant, Λ, also was computed from a MCNP model with M−15 Iridium−192 source, was centered at the origin of an evacuated phantom in which a critical volume containing air at STP was added 100 cm from the source center. The reference dose rate, $\dot{D}(r_{0},\theta_{0})\equiv \dot{D}(1\text{cm},\pi /2)$, is found to be $4.038\pm 0.064\;\text{cGy}\;{\text{mCi}}^{-1}\;h^{-1}$. The air kerma strength, $S_{K}$, is reported to be $3.632\pm 0.086\;\text{cGy}\;{\text{cm}}^{2}\;{\text{mCi}}^{-1}\;g^{-1}$, and the dose rate constant, Λ, is calculated to be $1.112\pm 0.029\;\text{cGy}\;h^{-1}\;U^{-1}$. The normalized dose rate, radial dose function, and anisotropy function with their uncertainties were computed and are represented in both tabular and graphical format in the report. A dosimetric study was performed of the new M−15 Iridium−192 HDR brachytherapy source using the MCNP6 radiation transport code. Dosimetric parameters, including the dose‐rate constant, radial dose function, and anisotropy function, were calculated in accordance with the updated AAPM and ESTRO dosimetric parameters for brachytherapy sources of average energy greater than 50 keV. These data therefore may be applied toward the development of a treatment planning program and for clinical use of the source.

PACS numbers: 87.56.bg, 87.53.Jw

## I. INTRODUCTION

Iridium−192 is a standard high‐dose‐rate brachytherapy isotope that has been well studied and tested for clinical treatments of lung, esophageal, prostate, cervical, coronary, and other diseases.^1^, ^2^, ^3^, ^4^, ^5^ Source Production & Equipment Co. (SPEC) has developed a new Iridium−192 high‐dose‐rate brachytherapy source model, the M‐15, for use in temporary high‐dose‐rate (HDR) brachytherapy. This model was designed to be an alternative replacement source for the Nucletron microSelectron Classic HDR afterloading system, since Nucletron no longer manufactures replacement sources for that system.

In accordance with the recommendation of the AAPM and ESTRO for brachytherapy sources with average energies greater than 50 keV^(6)^ and in accordance with the updated AAPM Task Group Report No. 43 (TG‐43U1),^(7)^ this report performed a complete series of Monte Carlo simulations to obtain the dosimetric parameters of the model M−15 Iridium−192 brachytherapy source. This was achieved using the MCNP6 Monte Carlo code^(8)^ to obtain dosimetric data in both water and air/vacuum modeled environments; the resulting data used to calculate source dosimetric parameters at one‐degree increments from 0° to 10° and 170° to 180° and five‐degree increments from 10° to 170°, as recommended by AAPM and ESTRO.^(6)^ The statistical uncertainties in each parameter also were calculated using the methods derived by Medich et al.^(9)^ and are presented herein.

## II. MATERIALS AND METHODS

### A. Brachytherapy source geometry and composition

**Figure 1:**
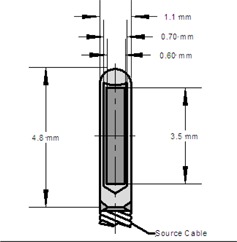
Configuration of the M−15 Iridium−192 HDR brachytherapy source modeled for the MCNP simulation. This model consists of a 3.5 mm long Iridium−192 source encapsulated in a steel shell.

The methods employed in this investigation of the SPEC model M−15 source were similar to the methods used previously for the SPEC model M‐19 Iridium−192 source.^(10)^ To summarize, the M−15 source was modeled in an MCNP Monte Carlo environment using the physical source specifications provided by the manufacturer. In this case, the MCNP6 code was used to obtain these results. A graphical depiction of the M−15 schematic used in the Monte Carlo simulation is presented in Fig. 1, with its corresponding physical parameters listed in Table 1. The source construction was symmetric about the coaxial axis, but nonsymmetric about the traverse plane due to the addition of an attached source cable.

**Table 1:** Physical parameters of the M−15 Iridium−192 source used in the MCNP simulations.

*Component*	*Outside Diameter (mm)*	*Inside Diameter (mm)*	*Length (mm)*	*Density (mg/mm^3^)*	*Remarks*
Active	0.6	N.A	3.5	22.4	Iridium Metal
Encapsulation	1.1	0.7	4.8	7.8	Stainless Steel
Source Cable	1.1	N.A	2000	7.8	Stainless Steel

### B. Monte Carlo calculation techniques

Radiation transport calculations were performed on a Windows‐based (Microsoft, Redmond, WA) personal computer running the MCNP6 Monte Carlo computer code.^(8)^ Iridium−192 photons were uniformly generated inside the iridium core of the model M−15 with photon and secondary electron transport replicated using default MCPLIB04 photoatomic cross‐sectional tables supplied with MCNP6. Simulations were performed for both water and air/vacuum computer models with a total of $4\times 10^{9}$ sources photon history for each simulation. All simulations were performed in the photon and electron transport mode (Mode: p,e in the MCNP code) so that both primary photons and resulting secondary electrons were properly transported.^(11)^ The complete Iridium−192 photon spectrum, presented by Firestone and Ekström,^(12)^ was used in these calculations and the total uncertainty in the spectrum, $\sigma_{I\gamma }^{\text{relative}}=0.5\%$, calculated as an intensity‐weighted average of the uncertainty in each spectral line also presented by Firestone and Ekström.

Dosimetric data, including $\dot{D}(r,\theta ),g_{L}(r),F(r,\theta ),\theta_{\text{an}}(r)$, and ${\bar{\varphi }}_{\text{an}}$, and their statistical uncertainty(^9^) were calculated from the output of an MCNP computer model consisting of an M−15 source placed at the center of a spherical water phantom of 100 cm diameter; this diameter was chosen to approximate an infinite water phantom, allowing for full photon scattering conditions in the dosimetric regions of interest.^11^, ^13^ Dosimetric data were calculated at radial distances ranging from 0.5 to 15 cm in half centimeter increments and over angles ranging from 0° to 180°, using the MCNP6 F6 energy deposition tally, $R_{\text{tally}}(r,\theta )$, reported in units of $\text{MeV}\;g^{-1}\;{\text{photon}}^{-1}$.

The energy deposition tally output, $R_{\text{tally}}$(r,θ), was multiplied by the Iridium−192 photon yield, $I_{\gamma }=2.301\;\gamma \;{\text{Bq}}^{-1}\;s^{-1}$, to obtain the calculated dose deposited in the tally region per disintegration, $\dot{D}(r,\theta )$:

(1) $$\dot{D}(r,\theta )=R_{MonteCarlo}(r,\theta )I_{\gamma }.$$

This result, expressed in units of $\text{MeV}\;g^{-1}\;{\text{Bq}}^{-1}\;s^{-1}$ was converted into more conventional units through the relationship: 1 $\text{MeV}\;g^{-1}\;{\text{Bq}}^{-1}\;s^{-1}=2.13\times 103\text{cGy}\;{\text{mCi}}^{-1}\;h^{-1}$.^(10)^

The air‐kerma strength in free space, $S_{K}$, was calculated using the methods described previously.^(9^) Briefly, the M−15 Iridium−192 source was centered at the origin of an evacuated phantom in which a critical volume containing air at STP was added 100 cm from the source center. The MCNP6 *F4 tally $\left(\text{Mev}/{\text{cm}}^{2}\right)$ output from the air‐kerma simulation was multiplied by the respective air mass‐energy absorption coefficient, $\mu_{\text{en}}/p\;({\text{cm}}^{2}/g)$,^(14^) to obtain the air kerma per source photon $\left(\text{MeV}\;g^{-1}\;{\text{photon}}^{-1}\right)$. Equivalence is assumed between the air mass‐energy transfer coefficient and the air mass‐energy absorption coefficient due to negligible radiative energy losses.^(15)^ The X‐ray cutoff energy, δ, was chosen to be δ=10%keV;^(9,10^) photons within the critical target with energies less than or equal to δ were removed from the final air‐kerma rate calculation. Once filtered, air‐kerma strength in free space is found by multiplying the square of the source to volume distance by the resulting air‐kerma rate.

## III. RESULTS & DISCUSSION

### A. Dose rate and normalized dose‐rate distribution

The Monte Carlo tally output $R_{\text{tally}}$(r,θ) at each point of interest in the water phantom is converted to the dose rate $\dot{D}(r,\theta )$ using Eq. (1). This dose rate corresponds to the updated AAPM TG‐43 dose rate in water:

(2) $${\dot{D}}_{TG-43}(r,\theta )=S_{K}*\Lambda *\frac{G_{L}(r,\theta )}{G_{L}(r_{0},\theta_{0})}*g_{L}(r)*F(r,\theta )$$

The absolute uncertainty in $\dot{D}(r,\theta )$ was calculated as the quadrature sum of the relative simulation uncertainty, the relative uncertainty in the cross section tables, and the relative uncertainty in the photon yield, as presented in Eq. (3):

(3) $$\sigma_{\dot{D}}^{absolute}(r,\theta )=\sqrt{{\left(\sigma_{MonteCarlo}^{relative}(r,\theta )\right)}^{2}+{\left(\sigma_{cros\text{sec}tion}^{relative}\right)}^{2}+{\left(\sigma_{I_{{}^{\gamma }}}^{relative}\right)}^{2}}$$

The data were next corrected for the geometry effect by multiplying the dose rate, $\dot{D}(r,\theta )$, by the radial distance squared, r^2^. The resulting dose rate is then normalized to the reference dose in water, $\dot{D}(r_{0},\theta_{0})=4.038\pm 0.064\;{\text{cGy mCi}}^{-1}\;h^{-1}$, to obtain ${\dot{D}}_{N}(r,\theta )$:

(4) $${\dot{D}}_{N}(r,\theta )=\frac{\dot{D}(r,\theta )\cdot r^{2}}{\dot{D}(r_{0},\theta_{0})}$$

**Figure 2:**
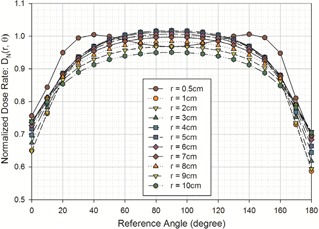
Presentation of the normalized dose‐rate distribution of model M−15 Iridium−192 from the MCNP output. Geometry effects were removed by multiplying the dose rate, $\dot{D}(r,\theta )$, by the radial distance squared, r^2^. Once corrected, the dose rate is then normalized to the dose rate at 1 cm along the seed's traverse's axis, $\dot{D}(r,\theta )=4.038\pm 0.064\;\text{cGy}\;{\text{mCi}}^{-1}\;h^{-1}$, to obtain the normalized dose rate.

A graphical representation of the normalized dose rate is presented in Fig. 2. The tabular representation is also presented in Table 2, with the absolute uncertainty calculated using the equation:

(5) $$\sigma_{{\dot{D}}_{N}}^{absolute}(r,\theta )={\dot{D}}_{N}(r,\theta )\sqrt{{\left(\sigma_{MonteCarlo}^{relative}(r,\theta )\right)}^{2}+{\left(\sigma_{MonteCarlo}^{relative}(r_{0,}\theta_{0})\right)}^{2}+2{\left(\sigma_{cross\text{sec}tion}^{relative}\right)}^{2}}$$

**Table 2:** Calculated normalized dose rate distribution of the M−15 Iridium−192 in water ${\dot{D}}_{N}(r,\theta )=[\dot{D}(r,\theta )\cdot r^{2}]\dot{D}(r_{0},\theta_{0})$ with absolute uncertainty, $\sigma_{{\dot{D}}_{N}}$.

r(cm)θ (deg)	*0.5*	*1.0*	*2.0*	*3.0*	*4.0*	*5.0*	*6.0*	*7.0*	*8.0*	*9.0*	*10.0*
0	0.756±0.016	0.650±0.014	0.648±0.014	0.674±0.015	0.698±0.015	0.716±0.015	0.727±0.016	0.736±0.016	0.740±0.016	0.740±0.016	0.738±0.016
1	0.760±0.016	0.655±0.014	0.654±0.014	0.676±0.014	0.698±0.015	0.716±0.015	0.730±0.016	0.739±0.016	0.744±0.016	0.745±0.016	0.742±0.016
2	0.760±0.016	0.656±0.014	0.664±0.014	0.688±0.015	0.709±0.015	0.725±0.015	0.738±0.016	0.747±0.016	0.751±0.016	0.752±0.016	0.749±0.016
3	0.761±0.016	0.667±0.014	0.677±0.014	0.699±0.015	0.720±0.015	0.735±0.016	0.747±0.016	0.754±0.016	0.758±0.016	0.759±0.016	0.755±0.016
4	0.764±0.016	0.681±0.014	0.690±0.015	0.712±0.015	0.730±0.015	0.745±0.016	0.757±0.016	0.764±0.016	0.766±0.016	0.765±0.016	0.761±0.016
5	0.775±0.016	0.696±0.015	0.703±0.015	0.723±0.015	0.741±0.016	0.755±0.016	0.765±0.016	0.771±0.016	0.773±0.016	0.772±0.016	0.767±0.016
6	0.789±0.017	0.710±0.015	0.716±0.015	0.734±0.016	0.751±0.016	0.764±0.016	0.773±0.016	0.779±0.017	0.781±0.017	0.778±0.017	0.773±0.016
7	0.803±0.017	0.723±0.015	0.727±0.015	0.746±0.016	0.761±0.016	0.774±0.016	0.782±0.017	0.787±0.017	0.787±0.017	0.785±0.017	0.779±0.017
8	0.817±0.017	0.736±0.016	0.741±0.016	0.758±0.016	0.773±0.016	0.785±0.017	0.792±0.017	0.796±0.017	0.796±0.017	0.793±0.017	0.786±0.017
9	0.830±0.018	0.750±0.016	0.753±0.016	0.769±0.016	0.783±0.017	0.794±0.017	0.801±0.017	0.804±0.017	0.804±0.017	0.800±0.017	0.792±0.017
10	0.844±0.018	0.764±0.016	0.767±0.016	0.782±0.017	0.795±0.017	0.805±0.017	0.811±0.017	0.813±0.017	0.811±0.017	0.807±0.017	0.799±0.017
15	0.906±0.019	0.827±0.018	0.825±0.018	0.836±0.018	0.845±0.018	0.851±0.018	0.853±0.018	0.852±0.018	0.847±0.018	0.840±0.018	0.829±0.018
20	0.950±0.020	0.875±0.019	0.870±0.018	0.878±0.019	0.884±0.019	0.887±0.019	0.886±0.019	0.883±0.019	0.876±0.019	0.866±0.018	0.854±0.018
25	0.978±0.021	0.910±0.019	0.904±0.019	0.910±0.019	0.914±0.019	0.915±0.019	0.912±0.019	0.907±0.019	0.899±0.019	0.888±0.019	0.874±0.019
30	0.995±0.021	0.936±0.020	0.930±0.020	0.934±0.020	0.937±0.020	0.936±0.020	0.933±0.020	0.926±0.020	0.917±0.019	0.904±0.019	0.890±0.019
35	1.002±0.021	0.955±0.020	0.950±0.020	0.953±0.020	0.955±0.020	0.954±0.020	0.949±0.020	0.941±0.020	0.931±0.020	0.918±0.019	0.902±0.019
40	1.005±0.021	0.968±0.021	0.966±0.020	0.969±0.021	0.969±0.021	0.967±0.021	0.962±0.020	0.954±0.020	0.943±0.020	0.929±0.020	0.913±0.019
45	1.003±0.021	0.978±0.021	0.978±0.021	0.981±0.021	0.981±0.021	0.978±0.021	0.973±0.021	0.964±0.020	0.952±0.020	0.938±0.020	0.922±0.020
50	0.999±0.021	0.986±0.021	0.987±0.021	0.990±0.021	0.990±0.021	0.987±0.021	0.981±0.021	0.972±0.021	0.960±0.020	0.946±0.020	0.929±0.020
55	0.994±0.021	0.991±0.021	0.995±0.021	0.998±0.021	0.998±0.021	0.995±0.021	0.988±0.021	0.979±0.021	0.967±0.021	0.952±0.020	0.935±0.020
60	0.988±0.021	0.994±0.021	1.001±0.021	1.004±0.021	1.004±0.021	1.000±0.021	0.994±0.021	0.984±0.021	0.972±0.021	0.957±0.020	0.940±0.020
65	0.983±0.021	0.997±0.021	1.005±0.021	1.008±0.021	1.008±0.021	1.005±0.021	0.998±0.021	0.989±0.021	0.976±0.021	0.961±0.020	0.943±0.020
70	0.977±0.021	0.998±0.021	1.008±0.021	1.012±0.021	1.012±0.021	1.009±0.021	1.002±0.021	0.992±0.021	0.979±0.021	0.964±0.020	0.946±0.020
75	0.974±0.021	0.999±0.021	1.011±0.021	1.015±0.022	1.015±0.022	1.011±0.021	1.004±0.021	0.994±0.021	0.982±0.021	0.966±0.020	0.949±0.020
80	0.970±0.021	0.999±0.021	1.012±0.021	1.016±0.022	1.016±0.022	1.013±0.021	1.006±0.021	0.996±0.021	0.983±0.021	0.968±0.021	0.950±0.020
85	0.969±0.021	1.000±0.021	1.013±0.021	1.017±0.022	1.017±0.022	1.013±0.021	1.007±0.021	0.997±0.021	0.984±0.021	0.969±0.021	0.951±0.020
90	0.968±0.021	*1.000*	1.013±0.021	1.017±0.022	1.017±0.022	1.014±0.022	1.007±0.021	0.997±0.021	0.984±0.021	0.969±0.021	0.951±0.020
95	0.969±0.021	1.000±0.021	1.013±0.021	1.017±0.022	1.017±0.022	1.013±0.021	1.007±0.021	0.997±0.021	0.984±0.021	0.969±0.021	0.951±0.020
100	0.971±0.021	0.999±0.021	1.012±0.021	1.016±0.022	1.016±0.022	1.013±0.021	1.006±0.021	0.996±0.021	0.983±0.021	0.968±0.021	0.950±0.020
105	0.973±0.021	0.999±0.021	1.011±0.021	1.015±0.022	1.015±0.022	1.011±0.021	1.004±0.021	0.994±0.021	0.982±0.021	0.966±0.020	0.948±0.020
110	0.978±0.021	0.999±0.021	1.009±0.021	1.012±0.021	1.012±0.021	1.009±0.021	1.002±0.021	0.992±0.021	0.979±0.021	0.964±0.020	0.946±0.020
115	0.983±0.021	0.997±0.021	1.006±0.021	1.009±0.021	1.009±0.021	1.005±0.021	0.999±0.021	0.989±0.021	0.976±0.021	0.961±0.020	0.943±0.020
120	0.989±0.021	0.995±0.021	1.001±0.021	1.004±0.021	1.004±0.021	1.001±0.021	0.994±0.021	0.984±0.021	0.972±0.021	0.957±0.020	0.940±0.020
125	0.995±0.021	0.991±0.021	0.995±0.021	0.998±0.021	0.998±0.021	0.995±0.021	0.988±0.021	0.979±0.021	0.967±0.021	0.952±0.020	0.935±0.020
130	1.000±0.021	0.987±0.021	0.988±0.021	0.991±0.021	0.991±0.021	0.988±0.021	0.982±0.021	0.972±0.021	0.960±0.020	0.946±0.020	0.929±0.020
135	1.004±0.021	0.979±0.021	0.979±0.021	0.981±0.021	0.981±0.021	0.979±0.021	0.973±0.021	0.964±0.020	0.952±0.020	0.938±0.020	0.921±0.020
140	1.006±0.021	0.969±0.021	0.966±0.020	0.969±0.021	0.970±0.021	0.967±0.021	0.962±0.020	0.954±0.020	0.943±0.020	0.929±0.020	0.913±0.019
145	1.004±0.021	0.955±0.020	0.950±0.020	0.953±0.020	0.955±0.020	0.953±0.020	0.948±0.020	0.941±0.020	0.930±0.020	0.917±0.019	0.902±0.019
150	0.996±0.021	0.936±0.020	0.929±0.020	0.933±0.020	0.935±0.020	0.935±0.020	0.931±0.020	0.925±0.020	0.915±0.019	0.903±0.019	0.888±0.019
155	0.978±0.021	0.909±0.019	0.902±0.019	0.907±0.019	0.911±0.019	0.912±0.019	0.909±0.019	0.904±0.019	0.896±0.019	0.885±0.019	0.871±0.018
160	0.948±0.020	0.870±0.018	0.864±0.018	0.872±0.018	0.878±0.019	0.881±0.019	0.881±0.019	0.877±0.019	0.871±0.018	0.861±0.018	0.849±0.018
165	0.897±0.019	0.815±0.017	0.812±0.017	0.823±0.017	0.833±0.018	0.839±0.018	0.842±0.018	0.841±0.018	0.838±0.018	0.831±0.018	0.821±0.017
170	0.810±0.017	0.730±0.015	0.734±0.016	0.752±0.016	0.768±0.016	0.780±0.017	0.788±0.017	0.792±0.017	0.792±0.017	0.788±0.017	0.782±0.017
171	0.788±0.017	0.708±0.015	0.717±0.015	0.737±0.016	0.754±0.016	0.767±0.016	0.776±0.016	0.780±0.017	0.781±0.017	0.779±0.017	0.773±0.016
172	0.763±0.016	0.686±0.015	0.698±0.015	0.720±0.015	0.738±0.016	0.752±0.016	0.762±0.016	0.768±0.016	0.770±0.016	0.769±0.016	0.764±0.016
173	0.733±0.016	0.664±0.014	0.678±0.014	0.701±0.015	0.722±0.015	0.738±0.016	0.749±0.016	0.756±0.016	0.760±0.016	0.759±0.016	0.755±0.016
174	0.713±0.015	0.643±0.014	0.659±0.014	0.684±0.015	0.706±0.015	0.723±0.015	0.736±0.016	0.744±0.016	0.749±0.016	0.749±0.016	0.746±0.016
175	0.703±0.015	0.626±0.013	0.642±0.014	0.669±0.014	0.692±0.015	0.711±0.015	0.724±0.015	0.733±0.016	0.739±0.016	0.740±0.016	0.738±0.016
176	0.694±0.015	0.613±0.013	0.629±0.013	0.656±0.014	0.679±0.014	0.699±0.015	0.714±0.015	0.723±0.015	0.730±0.015	0.731±0.016	0.730±0.015
177	0.692±0.015	0.603±0.013	0.617±0.013	0.645±0.014	0.669±0.014	0.689±0.015	0.704±0.015	0.715±0.015	0.723±0.015	0.725±0.015	0.724±0.015
178	0.694±0.015	0.592±0.013	0.606±0.013	0.634±0.013	0.660±0.014	0.680±0.014	0.697±0.015	0.708±0.015	0.716±0.015	0.718±0.015	0.718±0.015
179	0.692±0.015	0.591±0.013	0.596±0.013	0.622±0.013	0.649±0.014	0.671±0.014	0.688±0.015	0.700±0.015	0.708±0.015	0.710±0.015	0.711±0.015
180	0.692±0.015	0.587±0.013	0.594±0.013	0.618±0.013	0.644±0.014	0.664±0.014	0.684±0.015	0.696±0.015	0.702±0.015	0.708±0.015	0.705±0.015

### B. Geometry function, $G_{L}$(r,θ)

The geometry function, $G_{L}$(r,θ), accounting for the spatial distribution of radioactivity within the source, was calculated using the TG‐43U1 approximation for a line source of length *L* and subtended angle *β*:

(6) $$G_{L}(r,\theta )=\{\begin{array}{ll} \frac{\beta }{Lr\text{sin}(\theta )} & if\;\theta \neq 0^{0}\\ {\left(r^{2}-L^{2}/4\right)}^{-1} & if\;\theta =0^{0} \end{array}$$

### C. Radial dose function, $g_{L}$(r)

The radial dose function $g_{L}$(r) of model M−15 Iridium−192, a geometry independent dosimetric parameter accounting for dose falloff on traverse plane due to photon scattering and attenuation, was calculated using the TG‐43U1 formalism:

(7) $$g_{L}(r)=\frac{\dot{D}(r,\theta_{0})}{\dot{D}(r_{0},\theta_{0})}\frac{G_{L}(r_{0},\theta_{0})}{G_{L}(r,\theta_{0})}$$

The radial dose function was computed for radial distances from 0.25 cm to 10 cm. The absolute uncertainty $g_{L}(r)$ was also computed using Eq. (8):

(8) $$\sigma_{g_{L}}^{absolute}(r)=g_{L}(r)\sqrt{{\left(\sigma_{MonteCarlo}^{relative}(r,\theta_{0})\right)}^{2}+{\left(\sigma_{MonteCarlo}^{relative}(r_{0,}\theta_{0})\right)}^{2}+2{\left(\sigma_{cross\text{sec}tion}^{relative}\right)}^{2}}$$

**Figure 3:**
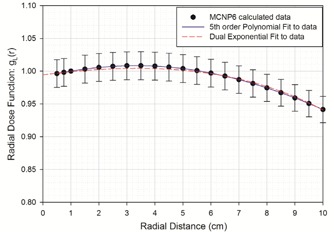
Calculated M−15 radial dose function, $g_{L}$(r), presented at distances between 0.5 and 10 cm. These data were fit to a 5th order polynomial, as recommended in TG‐43U1, and fit to a double exponential function, as discussed by Furhang and Anderson,^(16)^ for better accuracy when extrapolating outside of the measured data.

The resulting radial dose values are presented in Table 3. For clinical implementation, these results were fit to a 5th order polynomial. As the TG‐43U1^(7)^ recommended, polynomial fitting function is unreliable outside the distances for which data were obtained, a double exponential fit of the form $g_{L}(r)=C_{1}\cdot e^{\mu_{1}.r}+C_{2}\cdot e^{\mu_{2}.r}$ also is presented, since it better predicts the radial dose function outside our calculated data.^(16)^ The resulting 5th order polynomial coefficients and the double exponential coefficients are presented in Table 4. Graphical presentation of the computed results and fitted curves are presented in Fig. 3.

**Table 3:** Calculated radial dose function, $g_{L}$(r), for the M−15 Iridium−192 source with the absolute uncertainty, $\sigma_{\text{gL}}$.

r(cm)	$g_{L}(r)$	$\sigma_{gL}(r)$
0.5	0.996	0.021
1	1.000	0.000
2	1.006	0.021
3	1.008	0.021
4	1.008	0.021
5	1.004	0.021
6	0.997	0.021
7	0.987	0.021
8	0.974	0.021
9	0.959	0.020
10	0.941	0.020

**Table 4:** Summary of the 5th order polynomial regression coefficients and double exponential regression for the M−15 Iridium−192 radial dose function. For treatment planning, the radial dose function at r=0 should be derived from the double exponential fit resulting in $g_{L}(0)=0.994$.

*5th Order Polynomial Parameters*	*5th Order Polynomial Coefficients*	*Double Exponential Parameters*	*Double Exponential Coefficients*
$a_{0}$	0.9919	$C_{1}$	−0.08651
$a_{1}$	0.0092	$\mu_{1}$	0.1312
$a_{2}$	−0.0009	$C_{2}$	1.081
$a_{3}$	−0.0001	$\mu_{2}$	0.01549
$a_{4}$	$1.29\times 10^{-5}$		
$a_{5}$	$3.74\times 10^{-7}$		

### D. Anisotropy function, $F(r,\theta ),\phi_{\text{an}}(r)$, and ${\bar{\phi }}_{\text{an}}$

The anisotropy function, F(r,θ) was calculated using the TG‐43U1 equation:

(9) $$F(r,\theta )=\frac{\dot{D}(r,\theta )}{\dot{D}(r,\theta_{0})}\frac{G_{L}(r,\theta_{0})}{G_{L}(r,\theta )}$$

The uncertainty in the anisotropy function, F(r,θ) was calculated using the equation derived by Medich et al.:(^9^)

(10) $$\sigma_{F}^{absolute}(r,\theta )=F(r,\theta )\sqrt{{\left(\sigma_{MonteCarlo}^{relative}(r,\theta )\right)}^{2}+{\left(\sigma_{MonteCarlo}^{relative}(r_{0,}\theta_{0})\right)}^{2}+2{\left(\sigma_{cross\text{sec}tion}^{relative}\right)}^{2}}$$

**Figure 4:**
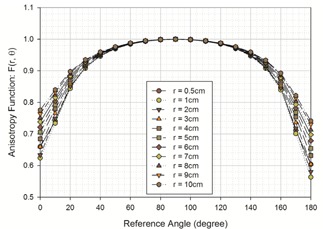
Resulting anisotropy function, F(r,θ), of the model M−15 source at angles between 0° and 180° and for depths between 0.5 cm to 10 cm.

The results of these calculations are presented in Table 5 and Fig 4.

**Table 5:** Calculated anisotropy function, F(r,θ), and anisotrpy factor, $\phi_{\text{an}}(r)$, and their absolute uncertainties for M−15 Iridium−192.

r(cm) θ(deg)	*0.5*	*1*	*2.0*	*3.0*	*4.0*	*5.0*	*6.0*	*7.0*	*8.0*	*9.0*	*10.0*
0	0.660±0.014	0.624±0.014	0.633±0.014	0.659±0.014	0.684±0.015	0.705±0.015	0.721±0.016	0.738±0.016	0.751±0.016	0.763±0.016	0.776±0.017
1	0.665±0.014	0.630±0.013	0.641±0.014	0.664±0.014	0.684±0.015	0.707±0.015	0.724±0.015	0.743±0.016	0.753±0.016	0.766±0.016	0.779±0.017
2	0.663±0.014	0.633±0.013	0.648±0.014	0.670±0.014	0.696±0.015	0.712±0.015	0.728±0.016	0.753±0.016	0.759±0.016	0.775±0.017	0.779±0.017
3	0.661±0.014	0.642±0.014	0.661±0.014	0.685±0.015	0.707±0.015	0.727±0.015	0.742±0.016	0.753±0.016	0.772±0.016	0.783±0.017	0.789±0.017
4	0.668±0.014	0.649±0.014	0.670±0.014	0.696±0.015	0.715±0.015	0.732±0.016	0.749±0.016	0.768±0.016	0.779±0.017	0.791±0.017	0.800±0.017
5	0.674±0.014	0.667±0.014	0.684±0.015	0.706±0.015	0.723±0.015	0.742±0.016	0.760±0.016	0.773±0.016	0.779±0.017	0.791±0.017	0.810±0.017
6	0.690±0.015	0.679±0.015	0.695±0.015	0.716±0.015	0.734±0.016	0.752±0.016	0.763±0.016	0.783±0.017	0.792±0.017	0.800±0.017	0.810±0.017
7	0.701±0.015	0.696±0.015	0.712±0.015	0.729±0.016	0.747±0.016	0.762±0.016	0.774±0.017	0.788±0.017	0.798±0.017	0.808±0.017	0.821±0.017
8	0.714±0.015	0.706±0.015	0.724±0.015	0.744±0.016	0.759±0.016	0.774±0.016	0.785±0.017	0.797±0.017	0.805±0.017	0.816±0.017	0.831±0.018
9	0.729±0.015	0.720±0.015	0.736±0.016	0.753±0.016	0.767±0.016	0.784±0.017	0.792±0.017	0.807±0.017	0.818±0.017	0.825±0.018	0.831±0.018
10	0.740±0.016	0.734±0.016	0.749±0.016	0.765±0.016	0.780±0.017	0.793±0.017	0.804±0.017	0.815±0.017	0.824±0.017	0.832±0.018	0.840±0.018
15	0.801±0.017	0.796±0.017	0.807±0.017	0.818±0.017	0.829±0.018	0.838±0.018	0.846±0.018	0.854±0.018	0.860±0.018	0.867±0.018	0.872±0.019
20	0.848±0.018	0.844±0.018	0.851±0.018	0.859±0.018	0.867±0.018	0.874±0.019	0.879±0.019	0.885±0.019	0.889±0.019	0.894±0.019	0.898±0.019
25	0.884±0.019	0.880±0.019	0.885±0.019	0.891±0.019	0.896±0.019	0.901±0.019	0.905±0.019	0.909±0.019	0.913±0.019	0.916±0.019	0.919±0.020
30	0.911±0.019	0.908±0.019	0.911±0.019	0.915±0.019	0.919±0.020	0.923±0.020	0.926±0.020	0.929±0.020	0.931±0.020	0.933±0.020	0.935±0.020
35	0.932±0.020	0.929±0.020	0.931±0.020	0.934±0.020	0.937±0.020	0.940±0.020	0.942±0.020	0.944±0.020	0.946±0.020	0.947±0.020	0.949±0.020
40	0.948±0.020	0.946±0.020	0.947±0.020	0.950±0.020	0.951±0.020	0.953±0.020	0.955±0.020	0.956±0.020	0.958±0.020	0.959±0.020	0.960±0.020
45	0.961±0.020	0.959±0.020	0.960±0.020	0.962±0.020	0.963±0.020	0.964±0.021	0.966±0.021	0.967±0.021	0.968±0.021	0.968±0.021	0.969±0.021
50	0.971±0.021	0.970±0.021	0.970±0.021	0.971±0.021	0.972±0.021	0.973±0.021	0.974±0.021	0.975±0.021	0.975±0.021	0.976±0.021	0.976±0.021
55	0.979±0.021	0.978±0.021	0.979±0.021	0.979±0.021	0.980±0.021	0.981±0.021	0.981±0.021	0.982±0.021	0.982±0.021	0.983±0.021	0.983±0.021
60	0.985±0.021	0.984±0.021	0.985±0.021	0.986±0.021	0.986±0.021	0.987±0.021	0.987±0.021	0.987±0.021	0.988±0.021	0.988±0.021	0.988±0.021
65	0.990±0.021	0.990±0.021	0.990±0.021	0.990±0.021	0.991±0.021	0.991±0.021	0.991±0.021	0.992±0.021	0.992±0.021	0.992±0.021	0.992±0.021
70	0.993±0.021	0.994±0.021	0.994±0.021	0.994±0.021	0.995±0.021	0.995±0.021	0.995±0.021	0.995±0.021	0.995±0.021	0.995±0.021	0.995±0.021
75	0.996±0.021	0.996±0.021	0.997±0.021	0.997±0.021	0.997±0.021	0.997±0.021	0.997±0.021	0.997±0.021	0.998±0.021	0.997±0.021	0.997±0.021
80	0.998±0.021	0.998±0.021	0.999±0.021	0.999±0.021	0.999±0.021	0.999±0.021	0.999±0.021	0.999±0.021	0.999±0.021	0.999±0.021	0.999±0.021
85	0.999±0.021	1.000±0.021	0.999±0.021	1.000±0.021	1.000±0.021	1.000±0.021	1.000±0.021	1.000±0.021	1.000±0.021	1.000±0.021	1.000±0.021
90	1.000	1.000	1.000	1.000	1.000	1.000	1.000	1.000	1.000	1.000	1.000
95	1.000±0.021	1.000±0.021	0.999±0.021	1.000±0.021	1.000±0.021	1.000±0.021	1.000±0.021	1.000±0.021	1.000±0.021	1.000±0.021	1.000±0.021
100	0.998±0.021	0.998±0.021	0.999±0.021	0.999±0.021	0.999±0.021	0.999±0.021	0.999±0.021	0.999±0.021	0.999±0.021	0.999±0.021	0.999±0.021
105	0.996±0.021	0.996±0.021	0.997±0.021	0.997±0.021	0.997±0.021	0.997±0.021	0.997±0.021	0.997±0.021	0.997±0.021	0.997±0.021	0.997±0.021
110	0.993±0.021	0.994±0.021	0.994±0.021	0.995±0.021	0.995±0.021	0.995±0.021	0.995±0.021	0.995±0.021	0.995±0.021	0.995±0.021	0.995±0.021
115	0.990±0.021	0.990±0.021	0.991±0.021	0.991±0.021	0.991±0.021	0.991±0.021	0.992±0.021	0.992±0.021	0.992±0.021	0.992±0.021	0.992±0.021
120	0.985±0.021	0.985±0.021	0.986±0.021	0.986±0.021	0.987±0.021	0.987±0.021	0.987±0.021	0.987±0.021	0.988±0.021	0.988±0.021	0.988±0.021
125	0.979±0.021	0.978±0.021	0.979±0.021	0.980±0.021	0.980±0.021	0.981±0.021	0.981±0.021	0.982±0.021	0.982±0.021	0.982±0.021	0.983±0.021
130	0.972±0.021	0.970±0.021	0.971±0.021	0.972±0.021	0.973±0.021	0.974±0.021	0.974±0.021	0.975±0.021	0.976±0.021	0.976±0.021	0.977±0.021
135	0.962±0.020	0.960±0.020	0.961±0.020	0.962±0.020	0.963±0.020	0.965±0.021	0.966±0.021	0.967±0.021	0.967±0.021	0.968±0.021	0.969±0.021
140	0.949±0.020	0.947±0.020	0.948±0.020	0.950±0.020	0.952±0.020	0.953±0.020	0.955±0.020	0.956±0.020	0.957±0.020	0.958±0.020	0.959±0.020
145	0.933±0.020	0.930±0.020	0.932±0.020	0.934±0.020	0.937±0.020	0.939±0.020	0.941±0.020	0.943±0.020	0.945±0.020	0.946±0.020	0.948±0.020
150	0.913±0.019	0.908±0.019	0.910±0.019	0.914±0.019	0.918±0.019	0.921±0.020	0.924±0.020	0.927±0.020	0.929±0.020	0.932±0.020	0.933±0.020
155	0.885±0.019	0.879±0.019	0.883±0.019	0.888±0.019	0.893±0.019	0.898±0.019	0.902±0.019	0.906±0.019	0.910±0.019	0.913±0.019	0.915±0.019
160	0.847±0.018	0.839±0.018	0.845±0.018	0.853±0.018	0.861±0.018	0.868±0.018	0.874±0.019	0.880±0.019	0.885±0.019	0.889±0.019	0.893±0.019
165	0.793±0.017	0.784±0.017	0.794±0.017	0.806±0.017	0.817±0.017	0.827±0.018	0.836±0.018	0.843±0.018	0.851±0.018	0.857±0.018	0.863±0.018
170	0.711±0.015	0.701±0.015	0.718±0.015	0.736±0.016	0.753±0.016	0.768±0.016	0.782±0.017	0.793±0.017	0.804±0.017	0.814±0.017	0.822±0.017
171	0.691±0.015	0.684±0.014	0.705±0.015	0.724±0.015	0.740±0.016	0.757±0.016	0.771±0.016	0.788±0.017	0.792±0.017	0.800±0.017	0.810±0.017
172	0.669±0.014	0.660±0.014	0.682±0.015	0.705±0.015	0.726±0.015	0.744±0.016	0.756±0.016	0.773±0.016	0.785±0.017	0.791±0.017	0.810±0.017
173	0.645±0.014	0.640±0.014	0.663±0.014	0.687±0.015	0.707±0.015	0.727±0.015	0.746±0.016	0.763±0.016	0.772±0.016	0.783±0.017	0.800±0.017
174	0.626±0.013	0.618±0.013	0.643±0.014	0.671±0.014	0.693±0.015	0.712±0.015	0.731±0.016	0.748±0.016	0.759±0.016	0.775±0.016	0.789±0.017
175	0.615±0.013	0.600±0.013	0.628±0.013	0.656±0.014	0.679±0.014	0.703±0.015	0.721±0.015	0.738±0.016	0.753±0.016	0.758±0.016	0.779±0.017
176	0.608±0.013	0.590±0.013	0.611±0.013	0.636±0.014	0.660±0.014	0.685±0.015	0.706±0.015	0.724±0.015	0.733±0.016	0.750±0.016	0.768±0.016
177	0.600±0.013	0.577±0.012	0.607±0.013	0.631±0.013	0.659±0.014	0.678±0.014	0.699±0.015	0.719±0.015	0.733±0.016	0.741±0.016	0.758±0.016
178	0.607±0.013	0.571±0.012	0.593±0.013	0.621±0.013	0.643±0.014	0.671±0.014	0.692±0.015	0.714±0.015	0.727±0.015	0.733±0.016	0.758±0.016
179	0.610±0.013	0.572±0.012	0.589±0.012	0.610±0.013	0.637±0.014	0.668±0.014	0.681±0.015	0.704±0.015	0.714±0.015	0.733±0.016	0.747±0.016
180	0.604±0.013	0.563±0.012	0.580±0.013	0.604±0.013	0.632±0.014	0.654±0.014	0.678±0.015	0.698±0.015	0.713±0.015	0.730±0.016	0.741±0.016
$\phi_{\text{an}}(r)$	1.009±0.015	0.971±0.014	0.963±0.014	0.964±0.014	0.965±0.014	0.966±0.014	0.967±0.014	0.968±0.014	0.969±0.014	0.970±0.014	0.971±0.014

The anisotropy factor, $\phi_{\text{an}}(r)$ was also calculated using the formula:

(11) $$\phi_{an}(r)=\frac{\int_{0}^{\pi }\dot{D}(r,\theta )\text{sin}(\theta )d\theta }{2\dot{D}(r,\theta_{0})}$$

The absolute uncertainty in $\phi_{\text{an}}(r)$ was calculated^(9)^ as:

(12) (σφanabsolute)2(r)=[φan(r)−Δθ2]2[σRtallyrelative(r,θ0)]2+∑θ=0θ≠θ0π(D˙(r,θ)D˙(r,θ0)sin(θ)2Δθ)2[σRtallyrelative(r,θ)]2

These results are also presented in Table 5 for the radial distance from 0.5 cm to 10 cm. From this data, the anisotropy constant was calculated to be ${\bar{\varphi }}_{an}=0.969\pm 0.007$ by integrating $\varphi_{an}(r)$ between 1 cm and 10 cm using the weighting factor^(9)^ $w(r)=\frac{\frac{1}{r^{2}}}{\sum_{r=1}^{10}\frac{\Delta r}{r^{2}}}$. The absolute uncertainty in ${\bar{\varphi }}_{an}(r)$ was calculated using the equation:

(13) $${\left(\sigma_{{\bar{\varphi }}_{an}}^{absolute}\right)}^{2}=\sum_{r=1}^{10}{\left[w(r)\Delta r\right]}^{2}\sigma_{\varphi_{an}}^{2}(r)$$

### E. $\dot{D}(r_{0},\theta_{0}),S_{k}$, and Λ

The Monte Carlo calculated reference dose rate in water $\dot{D}(r_{0},\theta_{0}),\equiv \dot{D}(1\text{cm},\pi /2)$ was determined to be $\dot{D}(r_{0},\theta_{0})=4.038\pm 0.064\;{\text{cGy mCi}}^{-1}h^{-1}$. The absolute uncertainty in $\dot{D}(r_{0},\theta_{0})$ was calculated using Eq. (3).

The air‐kerma in free space, $S_{k}$ was reported to be $3.632\;{\text{cGy cm}}^{2}\;{\text{mCi}}^{-1}\;h^{-1}$. The absolute uncertainty in $S_{k}$ was calculated to be $\sigma_{S_{k}}^{\text{absolute}}=0.086\;{\text{cGy cm}}^{2}\;{\text{mCi}}^{-1}\;h^{-1}$ using the following equation:

(14) $$\sigma_{S_{k}}^{absolute}=S_{k}\sqrt{{\left(\sigma_{MonteCarlo}^{relative}\right)}^{2}+{\left(\sigma_{cross\text{sec}tion}^{relative}\right)}^{2}+{\left(\sigma_{I_{\gamma }}^{relative}\right)}^{2}}$$

From the above data, the dose rate constant can be calculated using:

(15) $$\Lambda =\frac{\dot{D}(r_{0},\theta_{0})}{S_{K}}$$

The reported dose rate constant *Λ* is $\Lambda =1.112\;cGy\;h^{-1}\;U^{-1}$. The absolute uncertainty has been calculated to be using the following $\sigma_{\Lambda }^{\text{absolute}}=0.029\;{\text{cGy h}}^{-1}\;h^{-1}U^{-1}$ using the following equation:

(16) $$\sigma_{\Lambda }^{absolute}=\Lambda \sqrt{{\left(\sigma_{R_{tally}}^{relative}(r_{0},\theta_{0})\right)}^{2}+{\left(\sigma_{R_{air\;\text{ker}ma}^{\delta =10}}^{relative}\right)}^{2}}$$

## IV. CONCLUSIONS

A dosimetric study of the M−15 Iridium−192 HDR brachytherapy source was performed using the MCNP6 radiation transport code. The dose rate constant, radial dose function, and anisotropy function were calculated in accordance with the updated AAPM and ESTRO dosimetric parameters for brachytherapy sources of average energy greater than 50 keV. These data, therefore, may be applied toward clinical use and toward the development of a treatment program.
